# Inebriates

**Published:** 1901-12-28

**Authors:** 


### INEBRIATES.

The report of Dr. Branthwaite, the inspector under the Inebriates Acts, shows that at the close of the year 1900 there were 20 retreats which had received licences, and that the accommodation available had increased from 276 to 359 in the course of the year. A point which should be noted is that there seems as yet to be no efficient provision for absolutely destitute inebriates, there being only eight beds available for women in this condition and not one for a male case throughout the kingdom. A considerable proportion of the patients in the retreats are "private patients" as they are called, that is patients who enter by simple contract, as opposed to the

"signed" patients over whom some efficient control can be exercised, and it is somewhat unsatisfactory to find that the average term of voluntary detention in these "private" cases amounted to barely four-and-a-half months per case, a period which is evidently quite insufficient to do more than break the evil habit for a time. Inebriates persist in thinking themselves cured when they have abstained for a few weeks, and thus it is often impossible to do much permanent good unless the patients can be induced to give their consent, as provided by the Act, to a definite period of detention. Of certified inebriate reformatories five are mentioned as having been in use, with an aggregate of 416 beds. This is a considerable increase over the numbers for the previous year. The London County Council is so far the only local authority which possesses a reformatory of its own, certified and in working order, although 15 county councils have made arrangements for the reception of cases committed from courts within their jurisdiction. During the year 1900 144 "committed" cases were received into the reformatories, and it is worth noting that about 27 per cent. of them were the subjects of mental defects, a condition which greatly complicates the problem of treatment and lessens the prospect of recovery. Great difficulties have been experienced, both at the retreats and the reformatories, in managing the refractory patients, and it is clear that to discharge such cases, which has unfortunately proved sometimes to be the only available way of dealing with them, is most demoralising, tending as it does to set a direct premium upon misbehaviour.

